# In situ formation of reduced graphene oxide structures in ceria by combined sol–gel and solvothermal processing

**DOI:** 10.3762/bjnano.7.174

**Published:** 2016-11-23

**Authors:** Jingxia Yang, Johannes Ofner, Bernhard Lendl, Ulrich Schubert

**Affiliations:** 1 Institute of Materials Chemistry, Vienna University of Technology, Getreidemarkt 9, 1060 Wien, Austria; 2 permanent address: College of Chemistry and Chemical Engineering, Shanghai University of Engineering Science, LongTeng Road 333, 201620 Shanghai, P. R. China; 3 Institute of Chemical Technologies and Analytics, Vienna University of Technology, Getreidemarkt 9, 1060 Wien, Austria

**Keywords:** ceria, CO oxidation, graphene oxide, sol–gel processing

## Abstract

Raman and IR investigations indicated the presence of reduced graphene oxide (rGO)-like residues on ceria nanoparticles after solvothermal treatment in ethanol. The appearance of such structures is closely related to cerium *tert*-butoxide as precursor and ethanol as solvothermal solvent. The rGO-like residues improve the catalytic CO oxidation activity. This was also confirmed by introduction of “external” graphene oxide during sol–gel processing, by which the rGO structures and the catalytic activity were enhanced.

## Introduction

Ceria (CeO_2_) has been widely studied as catalyst or catalyst support for redox reactions owing to its high oxygen storage and release capacity. It is mostly used together with other components, such as noble metals or transition metal oxides, such as NiO or Co_3_O_4_, because synergistic effects improve the catalytic properties. Graphene-modified CeO_2_ greatly enhances the performance in electrochemical devices (supercapacitors, fuel cells or batteries) [1–7] or (photo-)catalysts [8–16]. The property enhancements are mainly due to the charge transfer between graphene and CeO_2_. For the preparation of graphene–CeO_2_ composites external graphene oxide (GO) is usually added to the ceria precursor or pre-synthesized ceria particles followed by reduction to reduced graphene oxide (rGO) [1–16].

In a previous study, we have synthesized CeO_2_ [17] from cerium *tert*-butoxide by combined sol–gel and solvothermal processing. The kind of post-synthesis treatment of the gels proved to be crucial for the specific surface area, the Ce^3+^ proportion and, as a consequence, the CO oxidation activity of the obtained materials, which were composed of 3.5–5.5 nm ceria nanoparticles. CeO_2_ solvothermally treated with EtOH had the highest surface area and showed better CO oxidation activity than the hydrothermally treated samples. A weight loss of ca. 12% was observed in TGA after solvothermal treatment of the gels with ethanol. This was not the case after hydrothermal treatment (only ca. 4%). We interpreted the high weight loss in the former samples to the presence of residual organic groups partly originating from EtOH and possibly associated with the high Ce^3+^ proportion (12%). We now show that the organic residues contain graphene-like structures. Furthermore, external graphene oxide was introduced into the system, to investigate how the graphene-like structures influence the properties and structure of the ceria–graphene composites.

Ceria or its composites are often prepared by solvothermal synthesis using various alcohols. In some reports organic residues on the surface of the ceria particles were noticed, even if ceria was prepared from (NH_4_)_2_[Ce(NO_3_)_6_], and alkoxide or carboxylate groups were identified [18]. Graphene-like structures, however, were never mentioned.

## Experimental

### Synthesis of rGO-modified CeO_2_

The procedure for the preparation of the ceria–rGO composites was the same as previously reported for that of pure ceria [17] (or Co_3_O_4_-modified CeO_2_ [19]) with the difference that varying proportions of GO were added to the precursor mixture. Graphene oxide (GO) was synthesized by the modified Hummer method [20–21]. All steps involving Ce(O*t*-Bu)_4_ were carried out under moisture-free argon using standard Schlenk or glove box techniques.

Ce(O*t*-Bu)_4_ (5 mmol) was dissolved in 1,2-dimethoxyethane (10 mL), followed by the addition of acetaldoxime (10 mmol) and stirring for 30 min, addition of the surfactant F127 (0.025 mmol) and additionally stirring for 1 h. No water was added during this stage. Different proportions of GO (0–0.2 g) were then added. The mixture was stirred for 30 min, ultrasonically treated for at least 2 h and then deposited onto glass sheets (20 × 30 cm^2^), which had been cleaned with 10% KOH, isopropanol and acetone and dried at 100 °C. The deposited films were exposed to ambient humidity at room temperature for 24 h (for hydrolysis and condensation along with solvent evaporation). The solid films were then scraped off with a razor blade to get a gel powder. The gel from 5 mmol Ce(O*t*-Bu)_4_ was transferred into a 60 mL autoclave with 30 mL EtOH, which was sealed, heated to 200 °C for 6 h and then cooled to room temperature by means of cold water. The solid was separated by centrifugation, washed at least three times with EtOH and H_2_O and then dried at 105 °C overnight. The samples were named rGO(*x*)-CeO_2_ (rGO was used to indicate GO after solvothermal treatment), where *x* is the mass of added GO in grams. For the sake of consistency, the sample with organic residues, but without externally added rGO is thus labelled rGO(0)–CeO_2_.

### Characterization

Raman spectra and maps were collected on a Horiba Jobin Yvon Micro-Raman spectrometer (LabRam 800 HR) equipped with an integral Olympus BX 41 microscope (20× objective) and a Peltier-cooled CCD detector, using the 632 nm line of a HeNe laser (1.5 mW) for excitation. A 600 line grating was used for obtaining the Raman spectra. The Raman–Stokes spectra were recorded in the range of 2500–300 cm^−1^ at 1.3 cm^−1^ spectral resolution. The spectrograph was calibrated using the 520 cm^−1^ Raman band of a Si wafer. Raman mapping was performed using a 10× magnification objective and a 300 line grating; a 532 nm (frequency doubled Nd:YAG) DPSS laser was used. An area of 500 × 500 µm^2^ with a lateral resolution of 5 µm was mapped by scanning each pixel three times for 1 s.

X-ray powder diffraction (XRD) measurements were performed on a Philips X'Pert diffractometer using Cu Kα radiation (λ = 1.5406 Å). High-resolution transmission electron micrographs (HRTEM) were recorded on a TECNAI F20 operated at 200 kV. Before the measurements, the samples were ultrasonically dispersed in EtOH for 30 min, and then deposited on copper grids covered with carbon films. FTIR spectra with 4 cm^−1^ resolution were recorded on a Bruker Tensor 27 equipped with an ATR Micro Focusing MVP-QL with a ZnSe crystal, using OPUS 4.0 software for analysis.

Thermogravimetric analysis (TGA) was performed on a Netzsch Iris TG 209 C in a platinum crucible in synthetic air with a heating rate of 10 °C/min. Nitrogen sorption measurements were performed on an ASAP 2020 (Micromeritics). The samples were degassed in vacuum at room temperature for at least 5 h prior to measurement. The total surface area was calculated according to Brunauer, Emmett and Teller (BET), and the pore size distribution (from the desorption branch) according to Barrett, Joyner and Halenda (BJH).

CO oxidation was performed in a continuous-flow fixed-bed quartz reactor under atmospheric pressure. A sample amount of 20 mg was loaded into the reactor and pretreated with synthetic air (30 mL/min) at 200 °C for 40 min (heating rate 10 °C/min). Then the sample was cooled to 30 °C in flowing synthetic air, and a mixture of 5 vol % CO, 10 vol % O_2_ and 85 vol % He (total flow 50 mL/min) was introduced. The system was then heated to 650 °C with a ramping rate of 5 °C/min. The concentrations of CO and CO_2_ in the outlet streams were monitored by gas chromatography with a HP-PLOT Q column and a flame ionization detector. For temperature-programmed reduction of CO (CO-TPR) the samples were exposed, after cooling, to a mixture of 5 vol % CO and 95 vol % He (total flow 50 mL/min) at room temperature. Then the system was ramped up to 900 °C at a heating rate of 10 °C/min. The gas stream was analyzed by an online quadrupole mass spectrometer (QMS) (Prisma Plus QMG 220, Pfeiffer Vacuum) equipped with a Faraday detector.

## Results and Discussion

**Figure 1:**
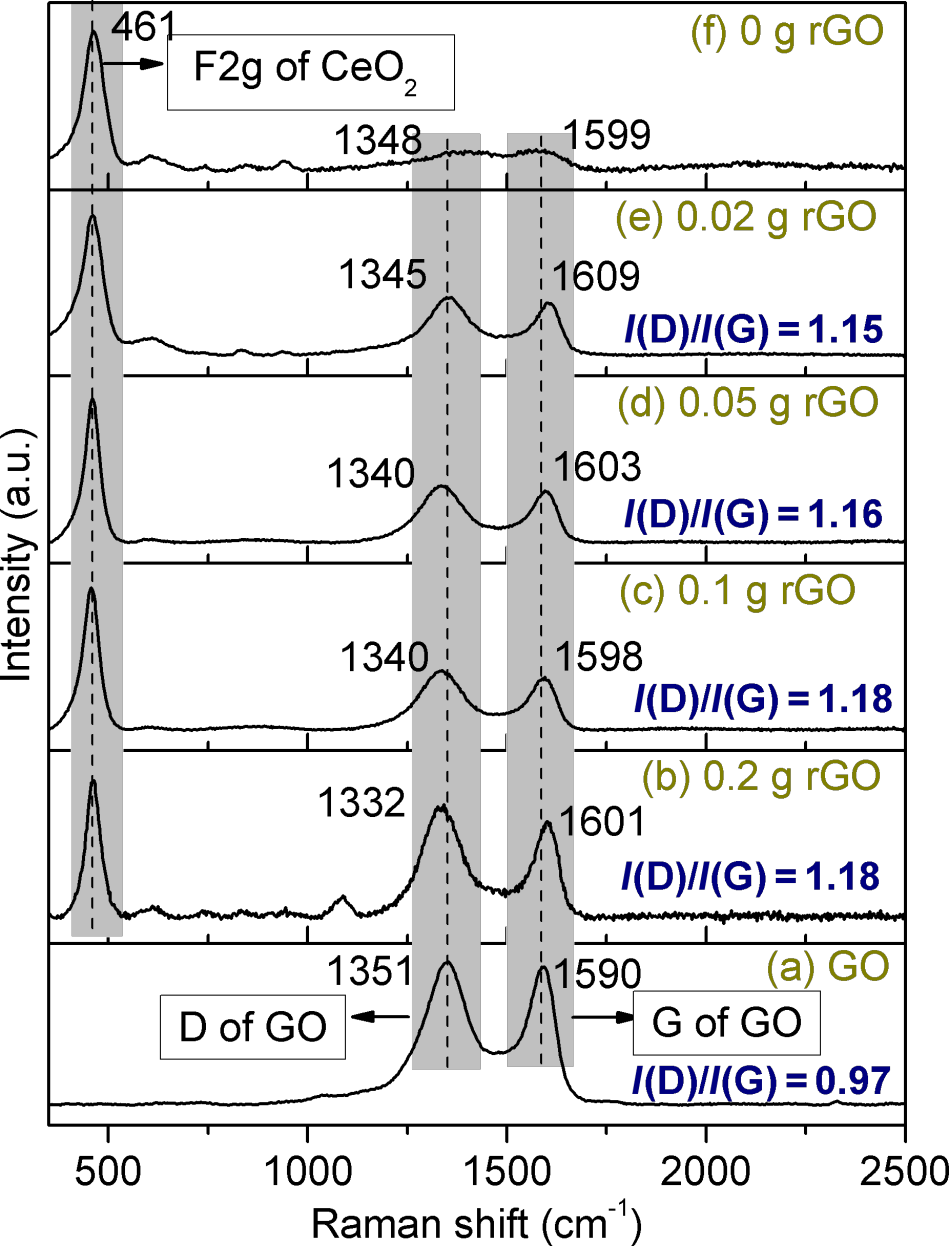
Raman spectra of GO (a); of CeO_2_ with rGO-like organic residues (sample rGO(0)–CeO_2_) (f); and of rGO–CeO_2_ composites with different proportions of added rGO (b–e) after solvothermal treatment in ethanol.

In order to shed light on the nature of the organic residues formed when the ceria gels were supercritically treated with ethanol [17], we performed extensive Raman studies. The sample (named rGO(0)–CeO_2_ according to the labelling scheme used in this article) surprisingly showed Raman bands that are attributed to the D- (1388 cm^−1^) and the G- (1577 cm^−1^) band of graphene (Figure 1f). As amorphous carbon shows no D-band [22–23], the organic residues have a graphene-like structure, indicating graphene-like structures in the organic residue. Furthermore, the F_2g_ band of CeO_2_ was also observed at 461 cm^−1^.

The D-band is related to a breathing mode of κ-point photons of A_1g_ symmetry, and the G-band can be attributed to the splitting of the E_2g_ stretching mode of graphite, which reflects the proportion of the sp^2^-hybridized carbon atoms [24]. The intensity ratio *I*(D)/*I*(G) represents the degree of disorder in a graphite layer. The GO synthesized in this study showed the typical D- and G-band at 1351 cm^−1^ and 1590 cm^−1^, respectively, with an intensity ratio *I*(D)/*I*(G) of 0.97. Generally, the G- and D-bands slightly shift to lower values when GO is reduced to graphene [25–26].

No D- and G-band were observed in the Raman spectra (Supporting Information File 1, Figure S1) when (1) H_2_O was used instead of EtOH for solvothermal treatment or (2) cerium ammonium nitrate (NH_4_)_2_[Ce(NO_3_)_6_] was used as precursor instead of cerium *tert*-butoxide (identical preparation conditions in all cases). We did not check cerium alkoxides, Ce(OR)_4_, with other groups R. This indicates that the appearance of residues with rGO-like structures is closely related to cerium *tert*-butoxide (or possibly Ce(OR)_4_ in general) as precursor and ethanol as the solvothermal solvent. The alcohol acts as a reductant for GO.

In this study, varying proportions of pre-synthesized graphene oxide (GO) were introduced into the ceria gel to investigate its dispersion and its effect on structure and properties of the sol–gel ceria. Raman spectra showed that the D-bands of all rGO–CeO_2_ composites were shifted to lower values (Figure 1), indicating that the GO had been reduced to graphene (rGO) during solvothermal processing in EtOH. The G-bands, however, were slightly shifted to higher values compared with that of GO. This can be attributed to the increased number of defects caused by stress from the oxygen states [16,27–28] as indicated by the intensity ratio *I*(D)/*I*(G). *I*(D)/*I*(G) values for all rGO–CeO_2_ composites were larger than that of GO, indicating that the number of defects in the graphene layer increased during the reduction of GO [29–30]. Anchoring of CeO_2_ on rGO also caused an intensity decrease and up-shifting of the G-band because of the electron transfer [9,16].

**Figure 2:**
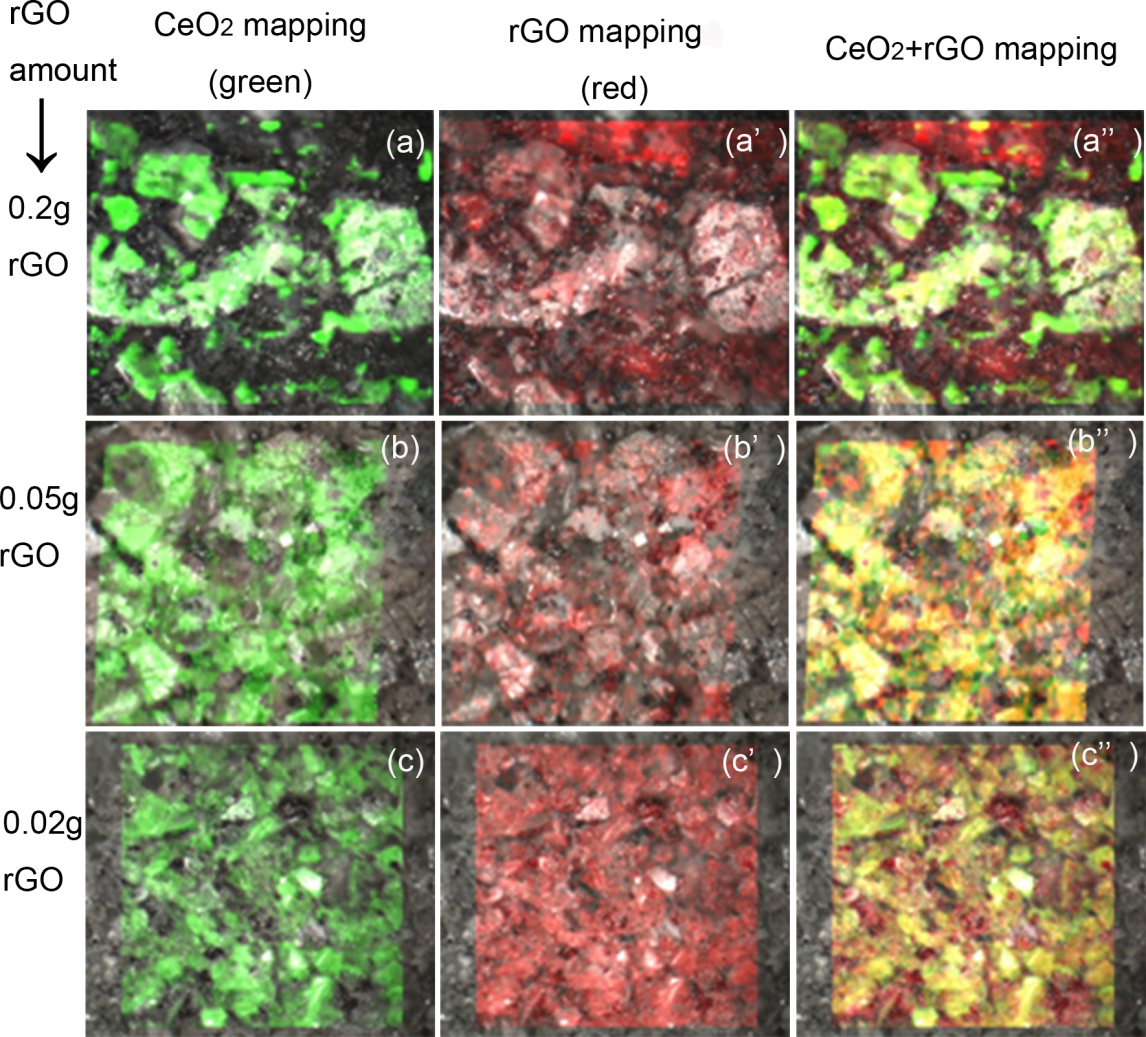
Raman mapping of rGO–CeO_2_ composites with different proportions of rGO after solvothermal treatment in ethanol (mapping area: 500 × 500 μm^2^).

The distribution of rGO was investigated by Raman mapping. Only the mappings for the samples with 0.2 g, 0.05 g and 0.02 g rGO are reproduced in Figure 2, because the Raman intensity for GO proportions of 0.1 and 0.05 g were almost the same. For a GO proportion of 0.05 and 0.02 g, the Raman mapping showed no obvious phase separation, indicating that rGO and CeO_2_ were homogeneously dispersed. However, when the GO proportion was 0.2 g, phase separation (agglomeration of rGO) was observed by partial disappearance of the CeO_2_ signal and the enhancement of the rGO signal in the corresponding area. In this case, GO apparently cannot disperse well enough after solvothermal treatment.

**Figure 3:**
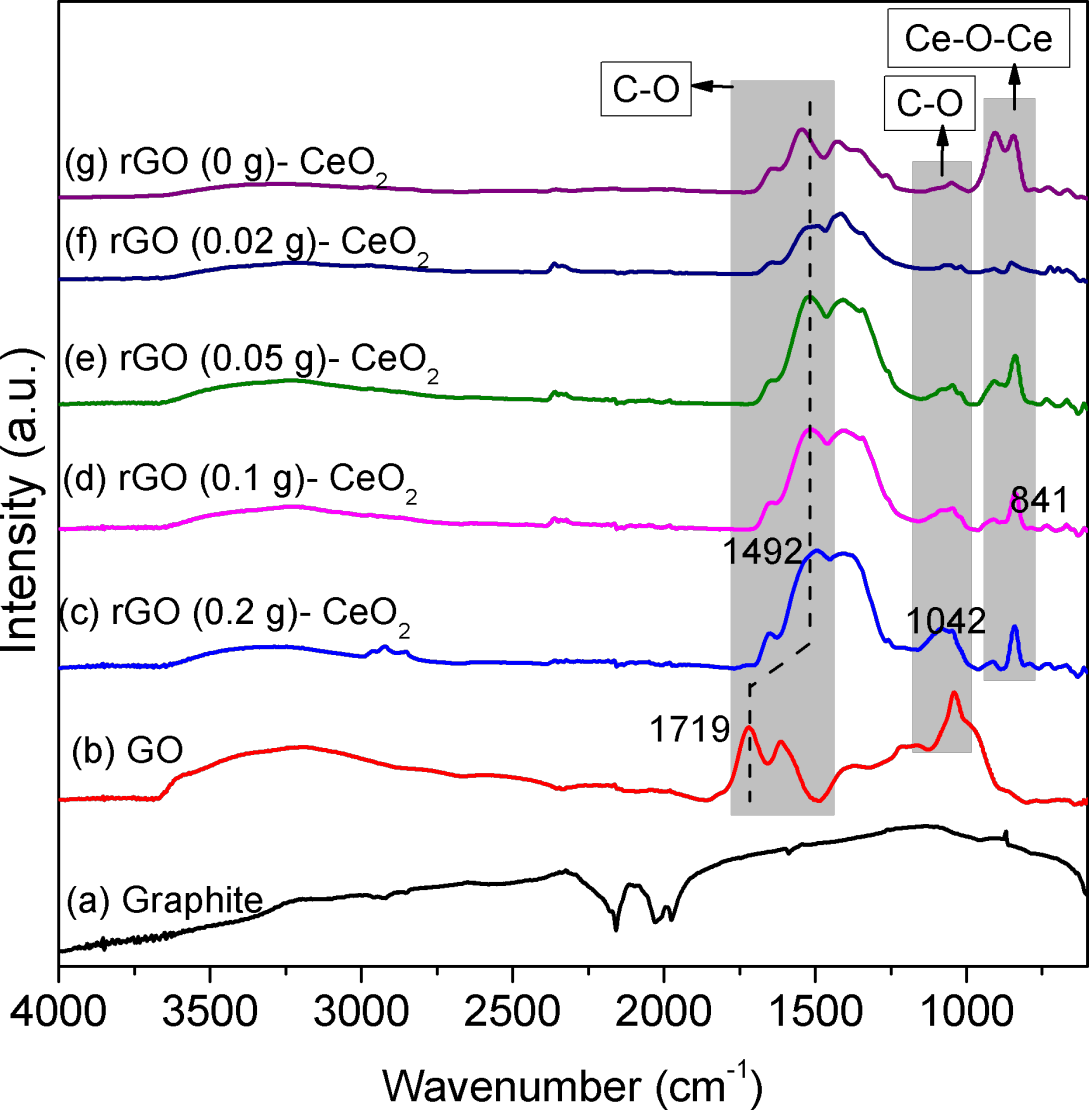
IR spectra of graphite (a); GO (b); CeO_2_ with rGO-like organic residues (sample rGO(0)–CeO_2_) (g); rGO–CeO_2_ composites with different proportions of added rGO (c–f) after solvothermal treatment in ethanol.

The IR spectra of all rGO–CeO_2_ composites (Figure 3) were almost the same, and only the peak intensities were somewhat different. They also showed a clear transition from GO to rGO. GO has strong bands in the range of 3000–3500 cm^−1^ and 1000–1750 cm^−1^, which correspond to OH and COO/CO groups, respectively. After the solvothermal treatment, the intensity of OH (3000–3500 cm^−1^) and C–O (1042 cm^−1^) vibrations decreased while the intensity of COO (1250–1700 cm^−1^) increased, indicating that part of the defects were repaired and rGO was formed. Compared with the COO groups of GO (Figure 3b), the positions of C=O bands shifted from 1719 cm^−1^ to 1492 cm^−1^. The shifts are most likely caused by coordination of graphene to CeO_2_ through the residual COO groups. The IR spectrum of the sample prepared without addition of rGO was similar to that of 0.02 g rGO. Part of the COO bands is most probably due to ceria-bound acetate groups, formed from ethanol either during the formation of CeO_2_ [18] or the reduction of GO. They cannot be distinguished spectroscopically from graphene-bound COO groups.

In the absence of externally added rGO, the TGA curve had only one shoulder at 150–250 °C, corresponding to a weight loss of 12.3%, as reported earlier [17]. The rGO-containing samples had an additional shoulder at 300–400 °C, which is probably due to the formation of rGO (Supporting Information File 1, Figure S2). The weight loss generally increased with increasing rGO proportion [12.4% for rGO(0.02)-CeO_2_, 16.5% for rGO(0.05)-CeO_2_, 19.1% for rGO(0.1)-CeO_2_, and 26.3% for rGO(0.2)-CeO_2_]. All weight losses were larger than the amount of added GO due to the organic residues after solvothermal treatment (ca. 11%). Thus, the formed rGO originates from both added GO and the organic residues.

Varying the proportion of GO (from 0 to 0.2 g) did not influence the CeO_2_ crystallite size (1.9–2.6 nm, calculated from Scherrer’s equation based on the strongest peak at 28.7°) to a large extent (Supporting Information File 1, Figure S3). The particle size of the undoped sample was 1.9 nm, and changed to 1.9–2.2 nm upon addition of 0.02–0.1 g GO. Only the sample rGO(0.2)–CeO_2_ had a slightly larger CeO_2_ crystallite size (2.6 nm).

**Figure 4:**
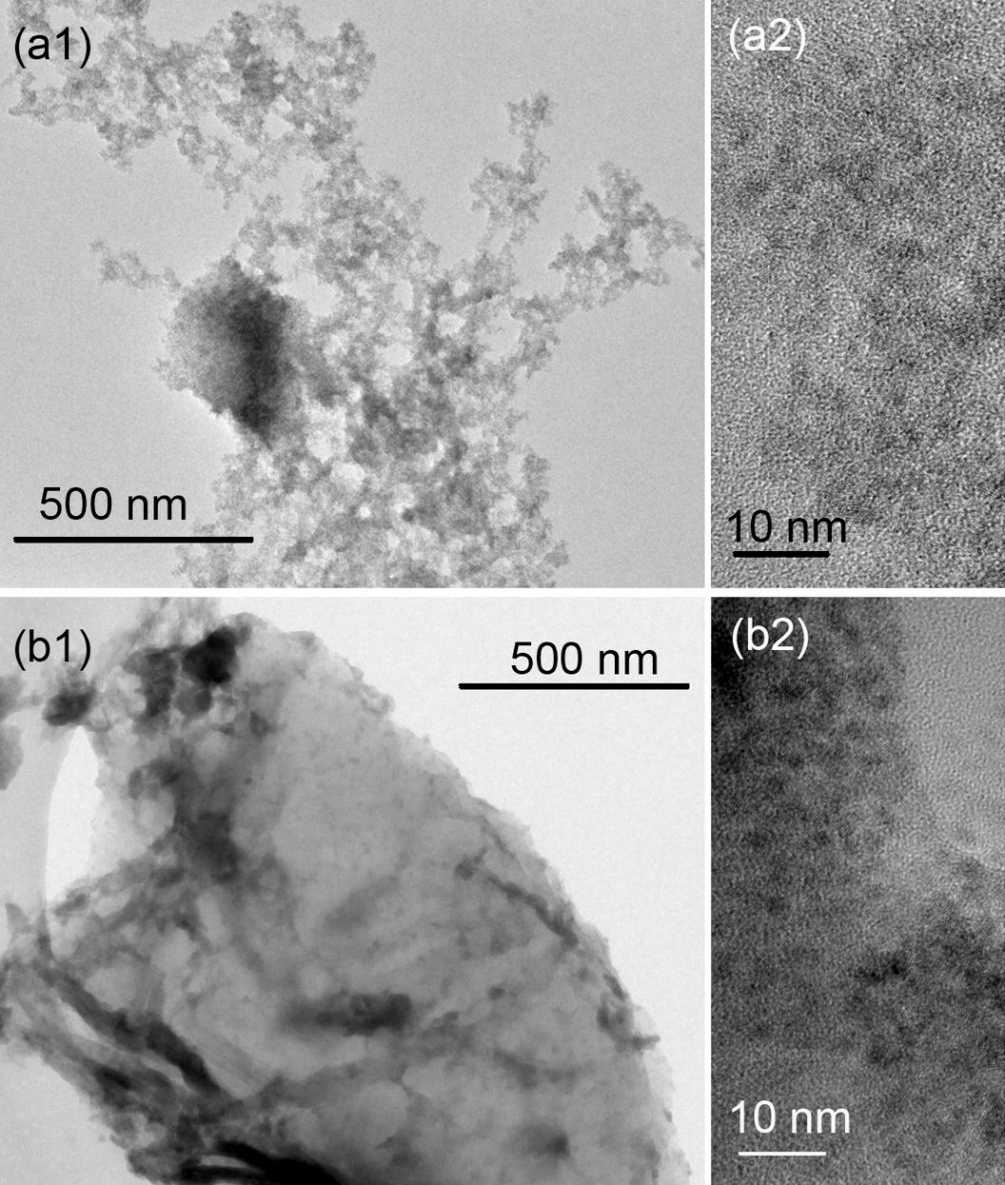
Morphologies of rGO(0)–CeO_2_ (a1: TEM, a2: HRTEM) and rGO(0.05)–CeO_2_ (b1: TEM, b2: HRTEM).

The TEM (Figure 4) were consistent with the XRD results and showed that rGO(0)–CeO_2_ and rGO(0.05)–CeO_2_ are formed by 2–4 nm ceria particles. The rGO sheets can be easily observed in rGO(0.05)–CeO_2_, and CeO_2_ particles were attached to the rGO sheets.

**Figure 5:**
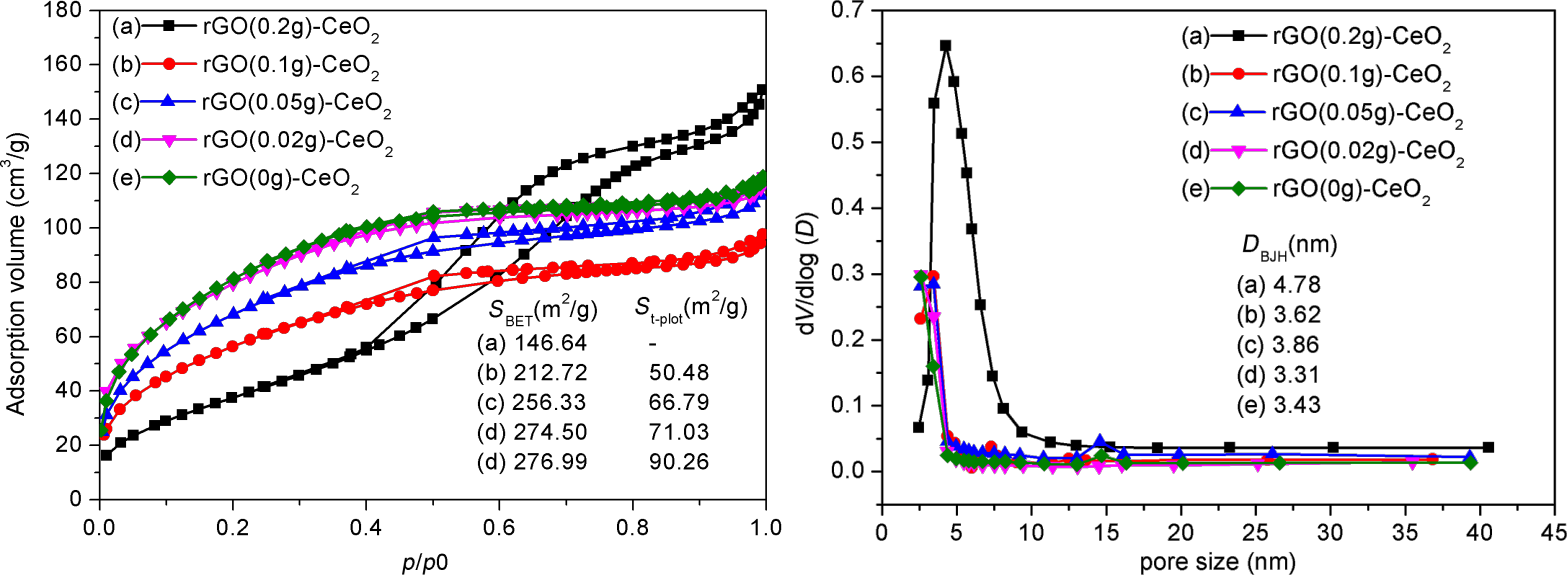
N_2_ adsorption–desorption isotherms (left) and pore size distributions (right) for CeO_2_ with rGO-like organic residues (rGO(0)–CeO_2_) (e) and rGO–CeO_2_ composites (a–d) with different proportions of rGO after solvothermal treatment with ethanol.

N_2_ adsorption–desorption results for the rGO–CeO_2_ composites with different proportions of rGO after solvothermal treatment are shown in Figure 5, left. Only rGO(0.2)–CeO_2_ is mesoporous, according to the IUPAC classification, while the others contain mainly micropores and a small portion of mesopores. This can also be seen from the pore size distribution (Figure 5, right). rGO(0.2)–CeO_2_ has an average pore size of 4.8 nm, while the other samples have smaller pore size in the range of 3.3–3.8 nm. The surface area increased with decreasing rGO proportion, from 147 m^2^/g with 0.2 g rGO to 275 m^2^/g with 0.02 g rGO. rGO(0)–CeO_2_ and rGO(0.02)–CeO_2_ processed almost the same surface area.

**Figure 6:**
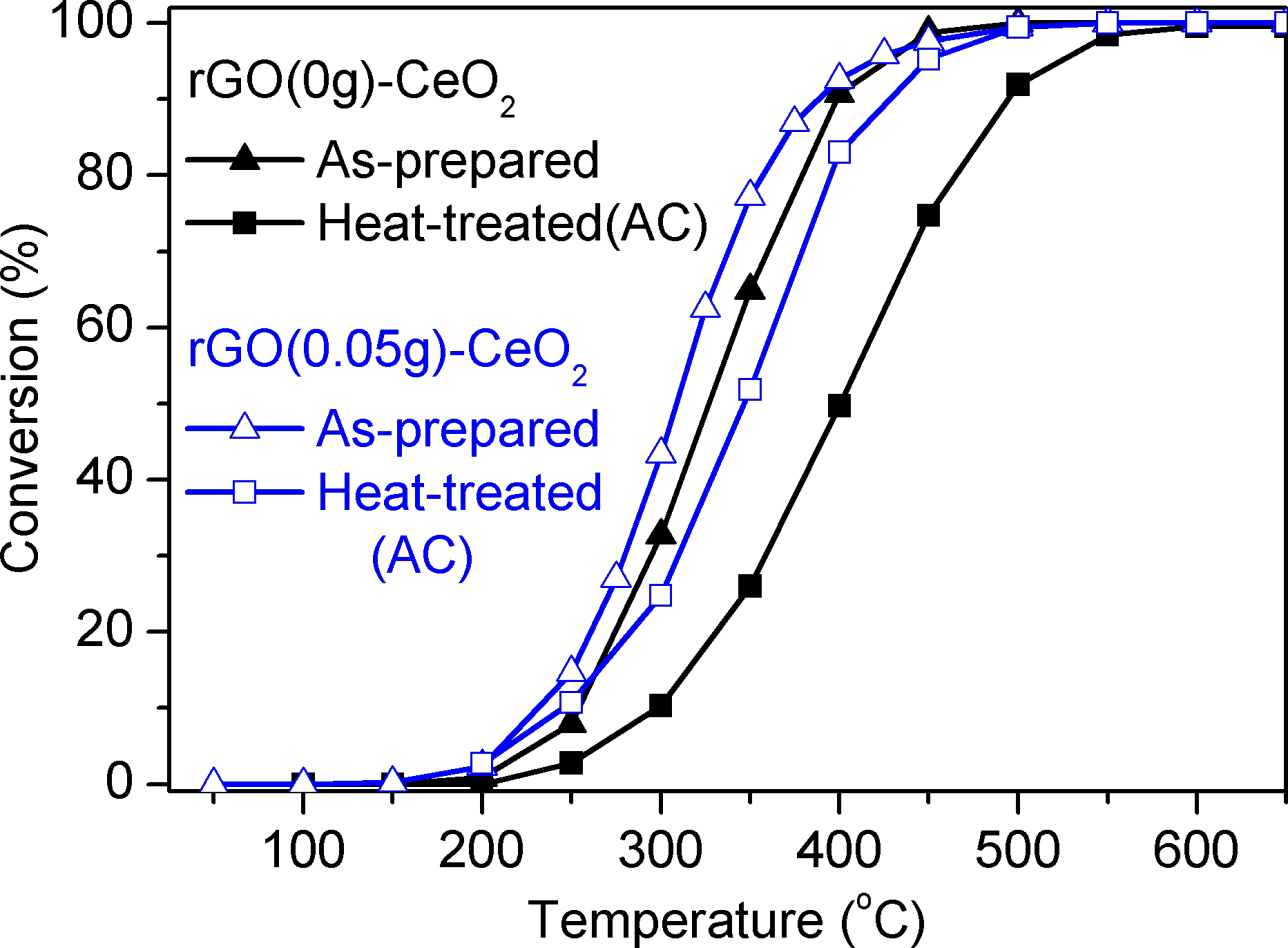
CO oxidation at different temperatures for CeO_2_ with rGO-like organic residues (rGO(0)–CeO_2_) and the rGO(0.05)–CeO_2_ composite.

The influence of the rGO proportion on the catalytic activity for CO oxidation was tested for rGO(0.05)–CeO_2_ compared to rGO(0)–CeO_2_ under the same conditions (Figure 6). The catalytic activity of rGO(0.05)–CeO_2_ was higher than that of rGO(0)–CeO_2_. Both samples were also calcined at 500 °C for 2 h to remove rGO and the organic residues (samples marked with AC). After calcination, the surface area of rGO(0.05)–CeO_2_ was reduced from 256.3 to 55.5 m^2^/g and that of rGO(0)–CeO_2_ from 276.9 to 88.9 m^2^/g. The activity of rGO(0.05)–CeO_2_–AC is somewhat lower than that of rGO(0.05)-CeO_2_ at the same temperature, but still higher than that of rGO(0)–CeO_2_–AC. For example, *r*_250 °C_ (the reaction rate at 250 °C per gram catalyst) for rGO(0.05)–CeO_2_ during first heating is 1.19·10^−5^ changed to 8.77·10^−6^ mol/s·g for rGO(0.05)–CeO_2_–AC. This decrease is possibly caused by the removal of graphene and reduction of the surface area.

**Figure 7:**
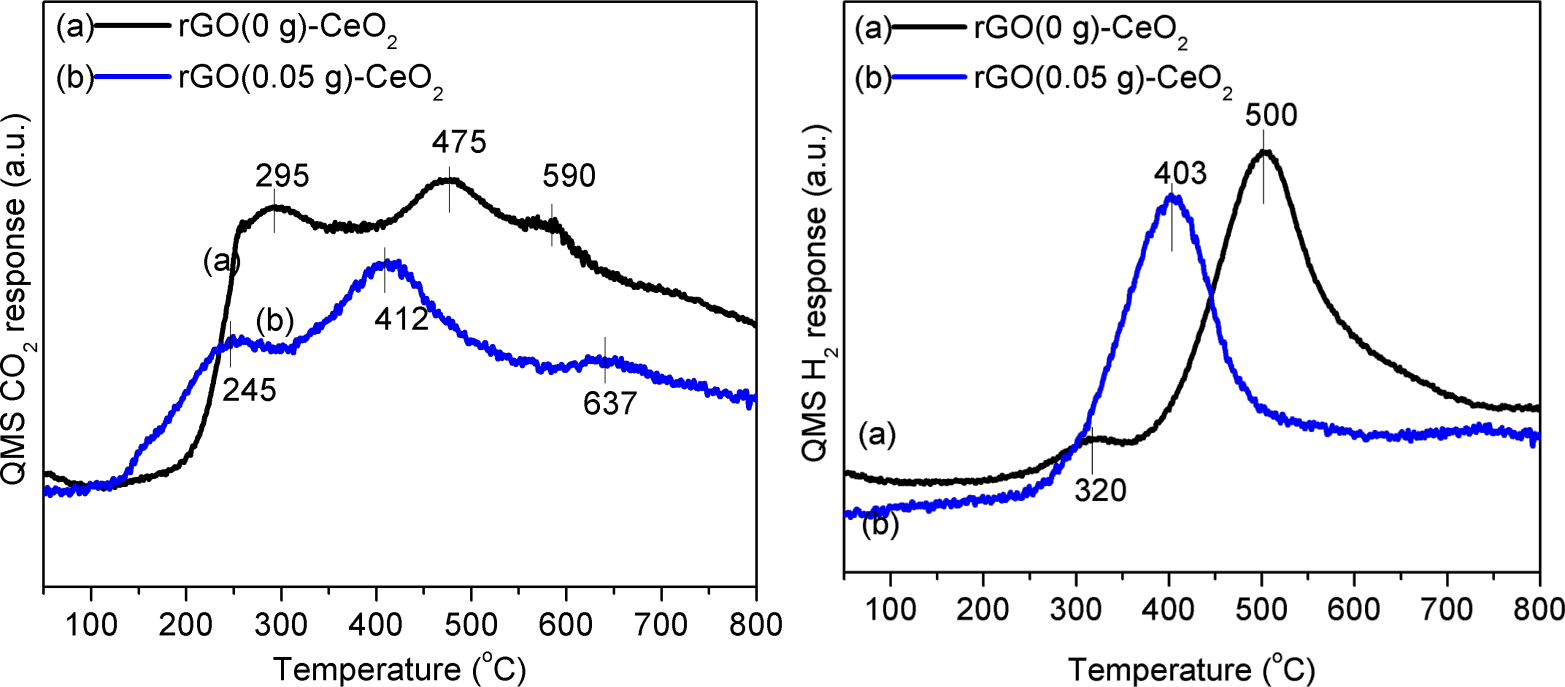
CO_2_ (left) and H_2_ (right) evolution during CO-TPR over rGO(0)–CeO_2_ and rGO(0.05)–CeO_2_.

The advantage of the rGO(0.05)–CeO_2_ composite can also be seen from the CO temperature-programmed reduction (CO-TPR) (Figure 7). Both CO_2_ and H_2_ evolution showed signals at lower temperatures (ca. 100 °C) for rGO(0.05)–CeO_2_ than for rGO(0)–CeO_2_. Similar to rGO(0)–CeO_2_, rGO(0.05)–CeO_2_ also showed three features: removal of surface lattice oxygen (below 300 °C), water-gas shift between CO and surface OH groups (300–500 °C), and extraction of bulk oxygen (above 500 °C), respectively, as discussed previously [17].

## Conclusion

We have shown in this article that the organic residues generated upon solvothermal treatment of ceria gels, obtained by sol–gel processing of cerium *tert*-butoxide, in ethanol as solvent [17] contain reduced graphene oxide (rGO)-like structures. The appearance of rGO-like structures can be associated with cerium *tert*-butoxide (or possibly cerium alkoxides in general) as precursor and ethanol as solvothermal solvent, and may also explain the higher catalytic activity (compared with, for example hydrothermally treated samples). This was also confirmed by introduction of “external” graphene oxide during sol–gel processing, by which the rGO structures and the catalytic activity were enhanced. The previously observed higher catalytic CO oxidation activity of ceria samples solvothermally treated in ethanol can therefore be traced back to the presence of rGO structures [17].

## Supporting Information

File 1 Additional experimental data.
