# Supplementary material for: In situ formation of reduced graphene oxide structures in ceria by combined sol–gel and solvothermal processing
Source: Beilstein J Nanotechnol. 2016 Nov 23;7:1815–21. doi: 10.3762/bjnano.7.174 (PMC5238676; doi:10.3762/bjnano.7.174)
Supplement: File 1 — Additional experimental data. [file Beilstein_J_Nanotechnol-07-1815-s001.pdf]

Supporting Information

for

In situ formation of reduced graphene oxide structures in ceria by combined sol–gel and solvothermal processing

Jingxia Yang[1,2], Johannes Ofner[3], Bernhard Lendl[3] and Ulrich Schubert*[1]

Address: [1]Institute of Materials Chemistry, Vienna University of Technology, Getreidemarkt 9, 1060 Wien, Austria; [2]permanent address: College of Chemistry and Chemical Engineering, Shanghai University of Engineering Science, LongTeng Road 333, 201620 Shanghai, P R. China and [3]Institute of Chemical Technologies and Analytics, Vienna University of Technology, Getreidemarkt 9, 1060 Wien, Austria

Email: Ulrich Schubert* - Ulrich.Schubert@tuwien.ac.at

* Corresponding author

Additional experimental data

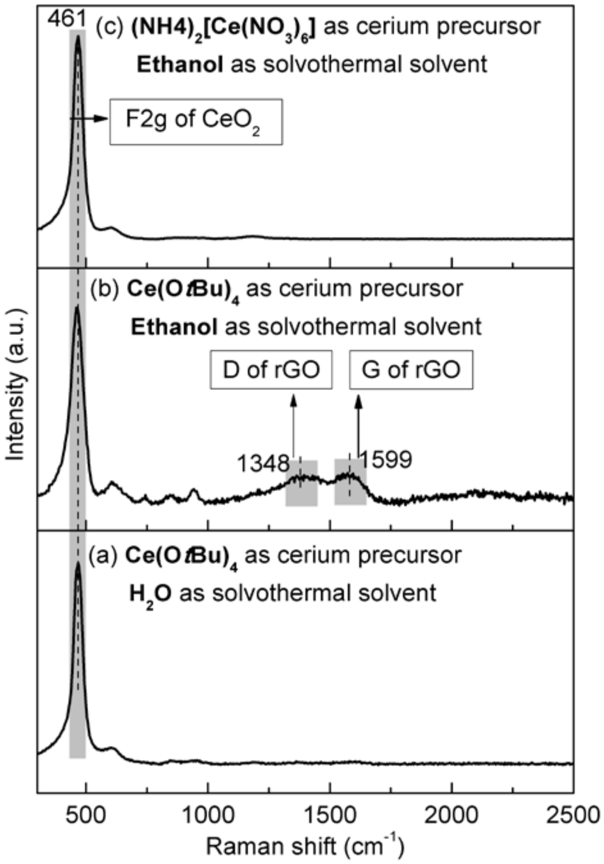

Figure S1: Raman spectra of ceria samples synthesized from different cerium precursors and different solvothermal solvents.

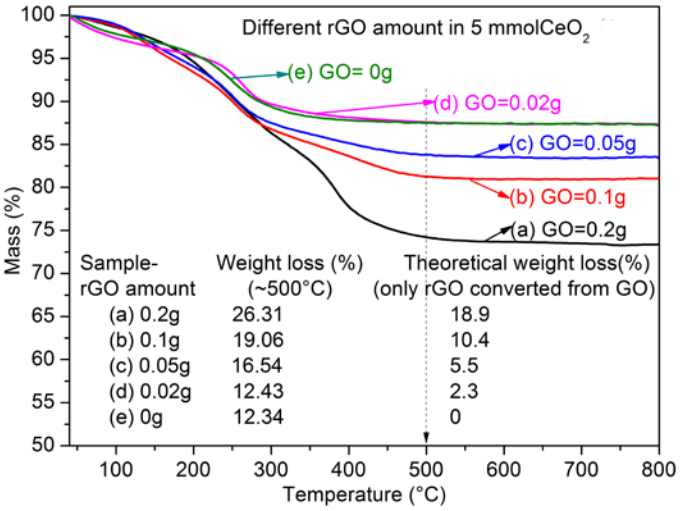

Figure S2: TGA curves of rGO–CeO_2 composites with different amounts of rGO after solvothermal treatment in ethanol.

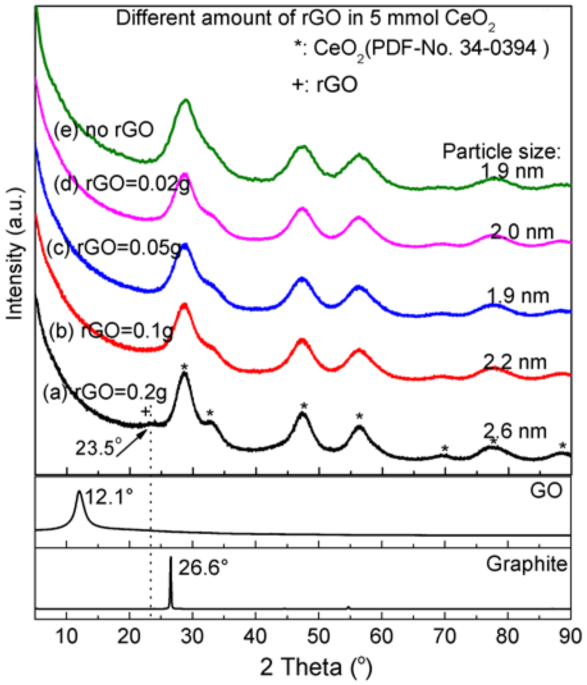

Figure S3: XRD patterns of graphite, GO and rGO–CeO_2 composites with different amounts of rGO amount after solvothermal treatment in ethanol.
